# Unfractionated heparin improves coagulation in sepsis by protecting glycocalyx of endothelia cells through inhibiting heparinase

**DOI:** 10.1515/jtim-2025-0094

**Published:** 2025-11-25

**Authors:** Lina Zhang, Jia Liu, Shengtian Mu, Hai Li, Yuxi Feng, Yan Wang, Yu Wang

**Affiliations:** Department of Critical Care Medicine, Liaoning Cancer Hospital & Institute, Shenyang, Liaoning Province, China; Shenyang Medical College, Shenyang, Liaoning Province, China

## To the editor

Sepsis is the most prevalent clinical syndrome in intensive care units (ICUs), with approximately one-third of sepsis patients developing organ insufficiency or failure and the reported mortality rates ranging from 50% to 70%.^[1]^ Endothelial injury plays a crucial role in the pathogenesis of sepsis by initiating both inflammatory and coagulation cascades, leading to microcirculatory dysfunction and subsequent organ failure.^[2,3]^ In the early stages of sepsis, the glycocalyx undergoes enzymatic degradation, compromising its protective functions and leading to increased such as anticoagulation and inhibition of cell adhesion resulting in enhanced vascular permeability, tissue edema, enhanced leukocyte adhesion, and further activation of coagulation pathways.^[4]^ Heparinase (HPA) is the only known mammalian glycosidase capable of degrading heparan sulfate (HS). In sepsis, inflammatory cells release mediators, activate latent HPA, resulting in enzymatic cleavage of HS and subsequent glycocalyx degradation.^[5,6]^ Unfractionated heparin (UFH) has been shown to protect the endothelial glycocalyx in sepsis. This study aimed to investigate whether UFH exerts its protective effects on the endothelial glycocalyx and inhibits coagulation activation in sepsis by inhibiting HPA.

A murine sepsis model was established using cecal ligation and perforation (CLP). Thirty male C57BL/6 mice (8–10 weeks old) were randomly assigned to five groups (*n* = 6 per group): control, CLP, CLP + UFH, CLP + HPA inhibitor, and CLP + UFH + HPA inhibitor. Mice in the treatment groups received an intraperitoneal injection of HPA inhibitor (20 mg/kg) before CLP and/or a tail vein injection of UFH (8 U/20 g) 1 h after CLP. The control and CLP groups received equivalent volumes of sterile saline. *In vitro*, human pulmonary microvascular endothelial cells (HPMECs) were treated with lipopolysaccharide (LPS) and divided into five groups: control, LPS, LPS + UFH, LPS + HPA, and LPS + UFH + HPA. Lung injury and pulmonary edema were assessed by histological staining and lung wet-to-dry weight ratio. Quantitative real-time polymerase chain reaction (qRT-PCR) was performed to analyze the expression levels of tissue factor (TF), fibrinogen (Fib), interleukin 1 β (IL-1β), syndecan-1, and HPA. Immunofluorescence staining and western blot analysis were conducted to assess syndecan-1 and HPA expression (Supplementary Materials). All procedures involving animals were carried out in compliance with the Guidelines for the Care and Use of Laboratory Animals and approved by the ethics committees of China Medical University (CMU2021066).

To evaluate the protective effects of UFH in sepsis, an *in vivo* CLP mouse model was established. Compared with the control group, the CLP group exhibited significant pathological changes, including widespread alveolar collapse and rupture, significant alveolar wall thickening, extensive infiltration of inflammatory granulocyte, interstitial and intra-alveolar hemorrhage, and significant fibrin deposition in the alveolar spaces, which confirmed the successful induction of sepsis (Supplementary Figure S1). In contrast, the CLP + UFH, CLP+HPA inhibitor, and CLP+UFH+HPA inhibitor groups exhibited significant improvements in lung histopathology compared with the CLP group. Furthermore, the CLP+UFH+HPA inhibitor group demonstrated less severe lung injury than that of the CLP+UFH or CLP+HPA inhibitor groups, with statistically significant difference (Supplementary Figure S2). Pulmonary vascular permeability was assessed by measuring lung wet-to-dry weight ratio and water content in mice from each group (Supplementary Figure S3). These findings suggest that CLP-induced HPA upregulation contributes to increased vascular permeability, and UFH, particularly in combination with HPA inhibition, effectively mitigates pulmonary edema. AT-III activity was significantly decreased in the CLP group compared with the control group (*P* < 0.001) (Supplementary Figure S4) indicating a hypercoagulable state typical of sepsis. Treatment with either UFH or the HPA inhibitor significantly increased circulating AT-III activity. Notably, the CLP+UFH+HPA inhibitor group exhibited a significantly greater increase in AT-III activity than the groups treated with UFH or the HPA inhibitor alone (*P* < 0.05), suggesting an additive effect of UFH and HPA inhibition in restoring anticoagulant function during sepsis.

**Figure 1:**
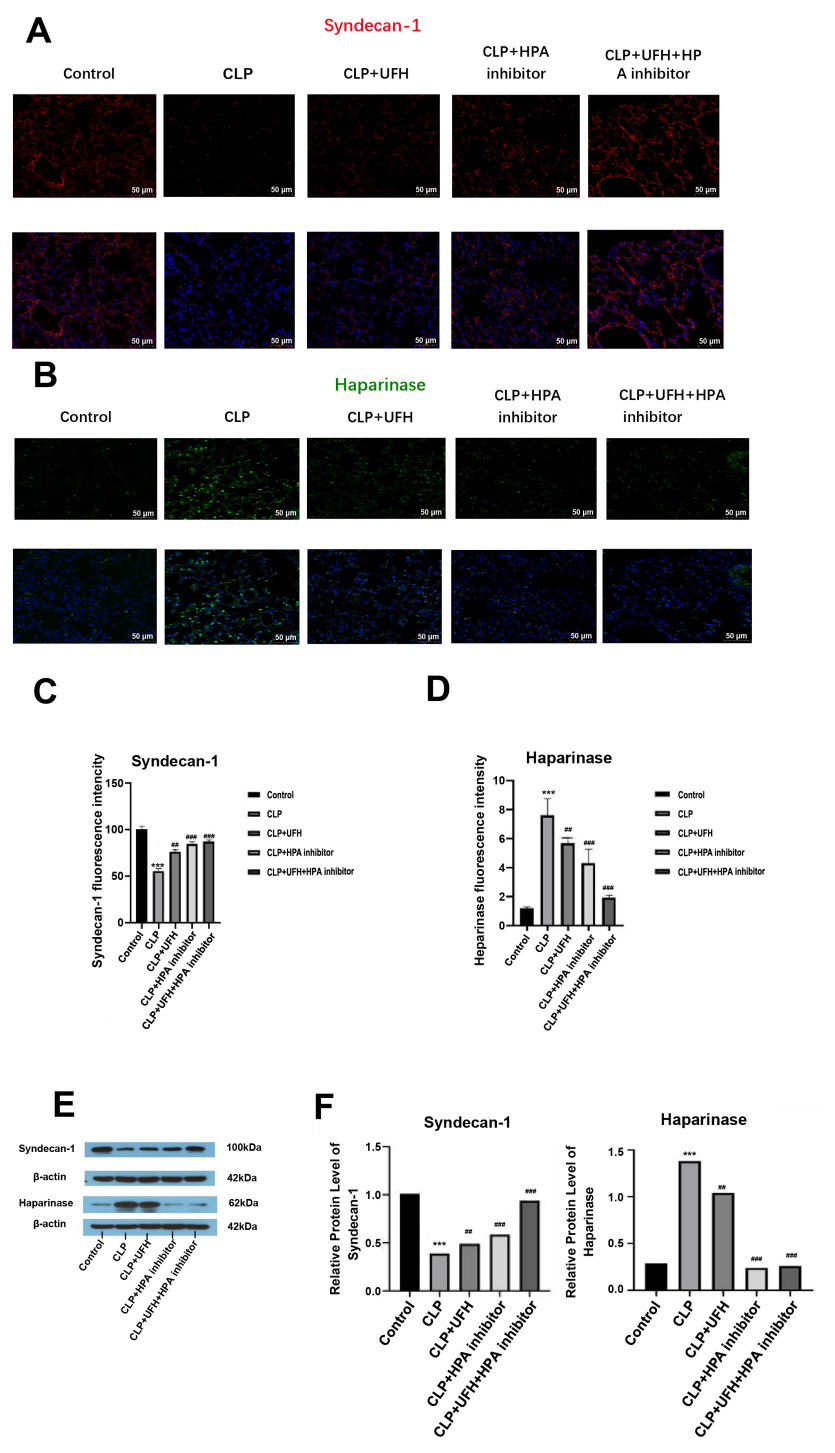
Expressions of syndecan-1 and heparinase in each group (*n* = 6, 200×). (A-D) Positive expression. Scale bar = 50 μm. (E, F) Relative expression. ***P < 0.001 *vs*. control group; ##*P* < 0.01, ###*P* <0.001 *vs*. CLP group. UFH, unfractionated heparin; HPA, heparinase. CLP, cecal ligation and puncture.

To further investigate the effects of UFH on coagulation and inflammatory responses during sepsis, the mRNA expression levels of TF, Fib, IL-1α and HPA were quantified in lung tissues (Supplementary Figures S5A–5C, 5E), while a decreased expression level of syndecan-1 (Supplementary Figures S5D). The protein expressions of syndecans-1 and HPA in lung tissues were assessed using immunofluorescence staining and western blotting. In the control group, immunofluorescence staining revealed a continuous and distinct staining pattern for syndecan-1, indicative of an intact glycocalyx (Figure 1A), while HPA staining was minimal (Figure 1B). In contrast, the CLP group showed a significant decrease in syndecan-1 expression, characterized by scattered fluorescence in the lung tissue, and a significant increase in HPA expression and widespread distribution, consistent with HPA-mediated glycocalyx degradation. In the UFH group, HPA protein expression was significantly decreased, while syndecan-1 was significantly increased, compared with the CLP group. These findings suggest that the protective effect of UFH on glycocalyx may be mediated by HPA inhibition, which was further confirmed by the increased fluorescence intensity of syndecan-1 observed in both the CLP+HPA inhibitor and CLP+UFH+HPA inhibitor groups. Notably, the decrease in HPA staining and increase in syndecan-1 expression was more significant in the CLP+UFH+HPA inhibitor group than in the CLP+UFH or CLP+HPA inhibitor groups (*P* < 0.01). Western Blot showed consistent results to that of immunostaining (Figure 1E, 1F).

To confirm the regulatory effects of UFH on coagulation and inflammation in sepsis *in vitro*, mRNA expression levels of TF and IL-1α were assessed in HPMECs following LPS stimulation (Supplementary Figures S6A and 6B). The results revealed a synergistic inhibitory effect of UFH and the HPA inhibitor on coagulation and inflammatory responses *in vitro*. The mRNA expression levels of syndecan-1 and HPA were also assessed to evaluate glycocalyx degradation *in vitro* (Supplementary Figures S6C and 6D). These findings support a synergistic protective effect of UFH treatment and HPA inhibition against LPS-induced glycocalyx degradation in HPMECs. Immunofluorescence staining showed that a synergistic protective effect of UFH and the HPA inhibitor on the glycocalyx and inhibit HPA expression following LPS stimulation (Supplementary Figures S7A-D). Western Blot showed consistent results to that of immunostaining (Supplementary Figures S8).

In sepsis, despite advancements in treatment strategies, including antibiotic therapy, mechanical ventilation, fluid resuscitation, and glycemic control, sepsis-related mortality remains significantly high.^[7]^ Studies suggest that increased inflammatory mediator levels, enhanced leukocyte adhesion, and activated coagulation pathways contribute to the development of multiple organ dysfunction.^[8, 9, 10]^ Endothelial glycocalyx degradation has been implicated in the pathogenesis of sepsis, contributing to vascular dysfunction, inflammation, and coagulation disorders. ^[8,9]^ UFH has been shown to protect the endothelial glycocalyx in sepsis. However, the mechanism is complex and unclear. This study aimed to investigate whether UFH exerts its protective effects on the endothelial glycocalyx and inhibits coagulation activation in sepsis by inhibiting HPA. *In vivo*, CLP induced lung injury and pulmonary edema, significantly increased TF, Fib, IL-1β, and HPA expression, and decreased syndecan-1 expression. Notably, treatment with UFH partially reversed these effects, and administration of the HPA inhibitor further enhanced the protective effects of UFH. *In vitro*, LPS stimulation of HPMECs resulted in increased TF, Fib, IL-1β, and HPA expression, and decreased syndecan-1 expression. UFH partially reversed these effects, with further improvements observed upon co-treatment with the HPA inhibitor. These results suggest that UFH exerts protective effects in sepsis by preserving the pulmonary endothelial glycocalyx, potentially by inhibiting the HPA-mediated degradation pathway. These findings highlight the therapeutic potential of targeting the HPA-glycocalyx axis in sepsis-induced vascular injury.

## Supplementary Information

Supplementary materials are only available at the official site of the journal (www.intern-med.com).

## Supplementary Material

Supplementary Material Details
