# Supplementary material for: Unfractionated heparin improves coagulation in sepsis by protecting glycocalyx of endothelia cells through inhibiting heparinase
Source: J Transl Int Med. 2025 Nov 25;13(6):618–21. doi: 10.1515/jtim-2025-0094 (PMC12721356; doi:10.1515/jtim-2025-0094)
Supplement: Supplementary file 1 — Supplementary Material Details [file jtim-2025-0094_sm.pdf]

Supplementary Table 1. Sequences of the Primers

Name	Primer sequence
TF	Forward: 5'-TACCTTACCGAGACACAAACCT-3'
	Reverse: 5'-CTTTCCCGTGCTTGAGCCTT-3'
FIB	Forward: 5'-GTACGTGGCCCAAGAGTTGT-3'
	Reverse: 5'-AAGGGCATTTGTGGTTCCAGT-3'
IL-1β	Forward: 5'-TGCCACCTTTTGACAGTGATG-3'
	Reverse: 5'-AAGGTCCACGGGAAAGACAC-3'
Syndecan-1	Forward: 5'-CCAGAGGAGACAGAGCCTAAC-3'
	Reverse: 5'-TGGTCCAGGGTTTCTTACTCC-3'
HPA	Forward: 5'-TGCCACCTTTTGACAGTGATG-3'
	Reverse:5'-AAGGTCCACGGGAAAGACAC-3'
GAPDH	Forward: 5'-GGTTGTCTCCTGCGACTTCA-3'
	Reverse: 5'-TGGTCCAGGGTTTCTTACTCC-3'

TF, tissue factors; FIB, fibrinogen; IL-1β: interleukin-1β; HPA, heparinase.

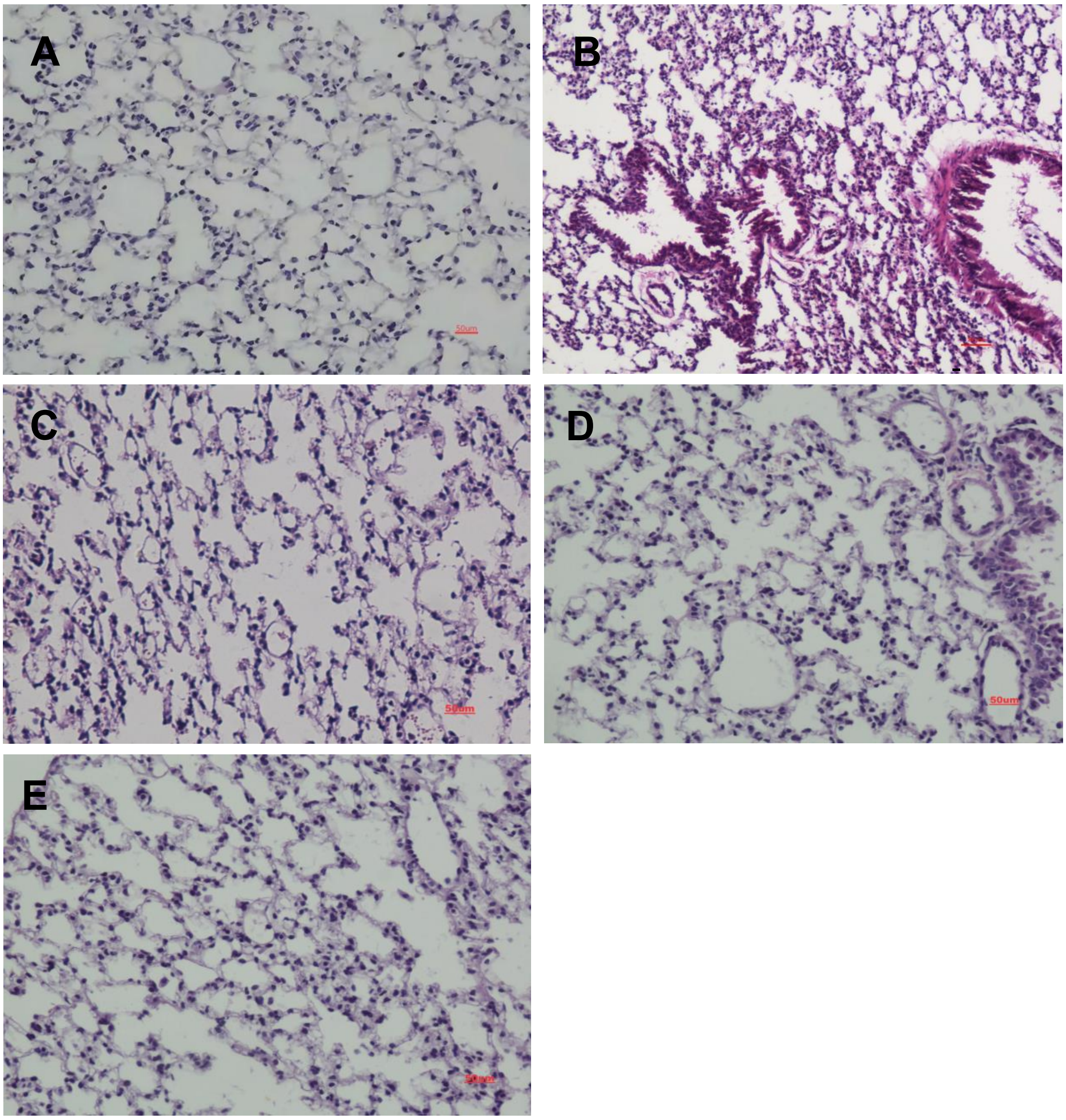

Supplemental Figure 1. Histological evaluation of lung was conducted by hematoxylin–eosin (HE) staining (200×, scale bar 50 μm). A. Control group; B. CLP group; C. CLP+UFH group; D. CLP+HPA inhibitor group; E. CLP+UFH+HPA inhibitor group. CLP, cecal ligation and puncture; UFH, unfractionated heparin; HPA, heparinase.

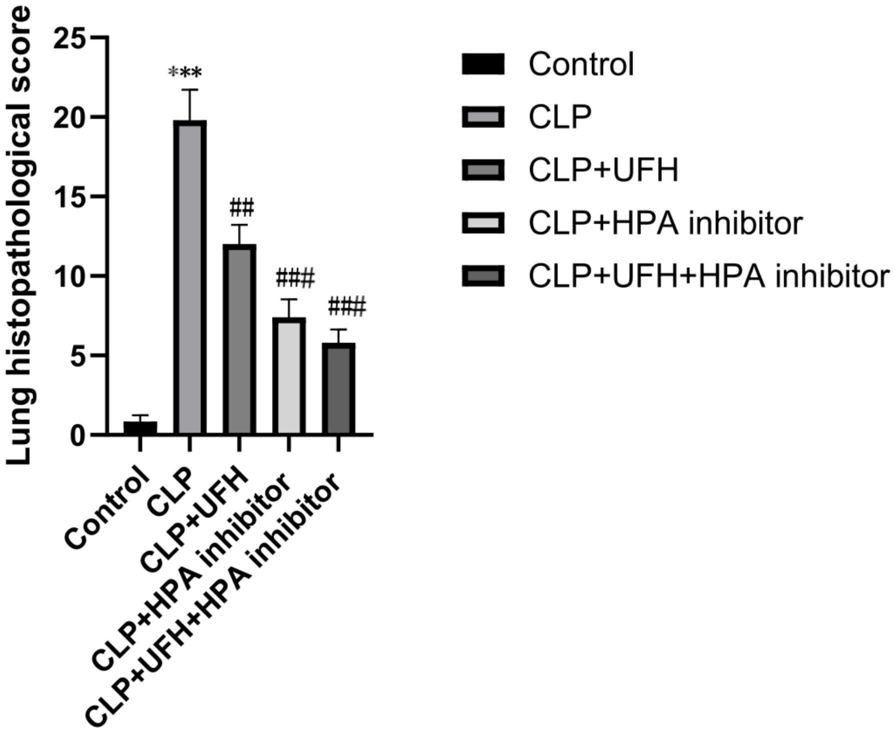

Supplemental Figure 2. Histological score of lung tissue in each group. The lung histopathological score in control group is 0-1. The lung histopathological score in CLP and CLP+UFH group are 19-23 and 10-14 respectively. The lung histopathological score in CLP+HPA inhibitor and CLP+UFH+HPA inhibitor group is 10-13 and 8-10 respectively. UFH, unfractionated heparin; HPA, heparinase. $N = 6$. $^{***}P < 0.001$ *vs*. control group; $^{\#\#}P < 0.01$, $^{\#\#\#}P < 0.001$ *vs*. CLP group.

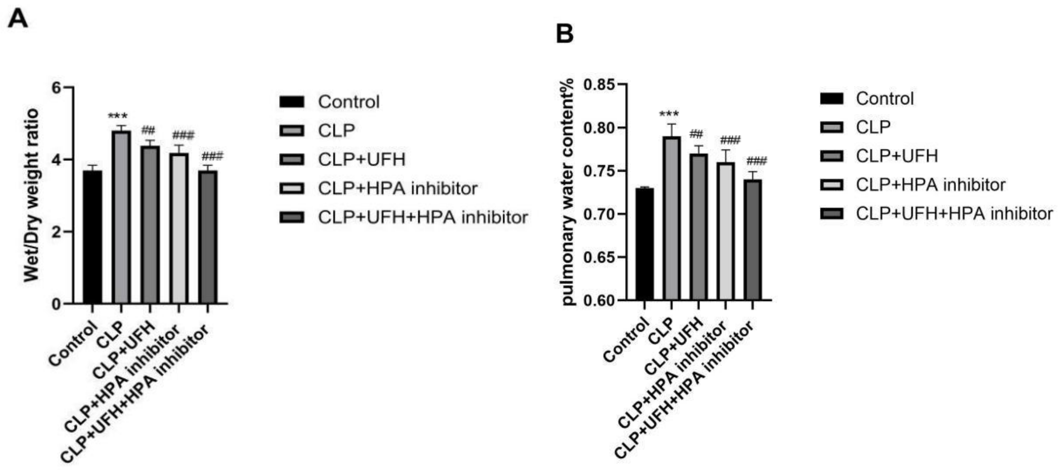

Supplemental Figure 3. Wet/Dry weight ratio. (A) and pulmonary water contents. (B) in each group. *N* = 6. $^{***}P < 0.001$ *vs.* control group; $^{\#\#}P < 0.01$, $^{\#\#\#}P < 0.001$ *vs.* CLP group. CLP, cecal ligation and puncture; UFH, unfractionated heparin; HPA, heparinase.

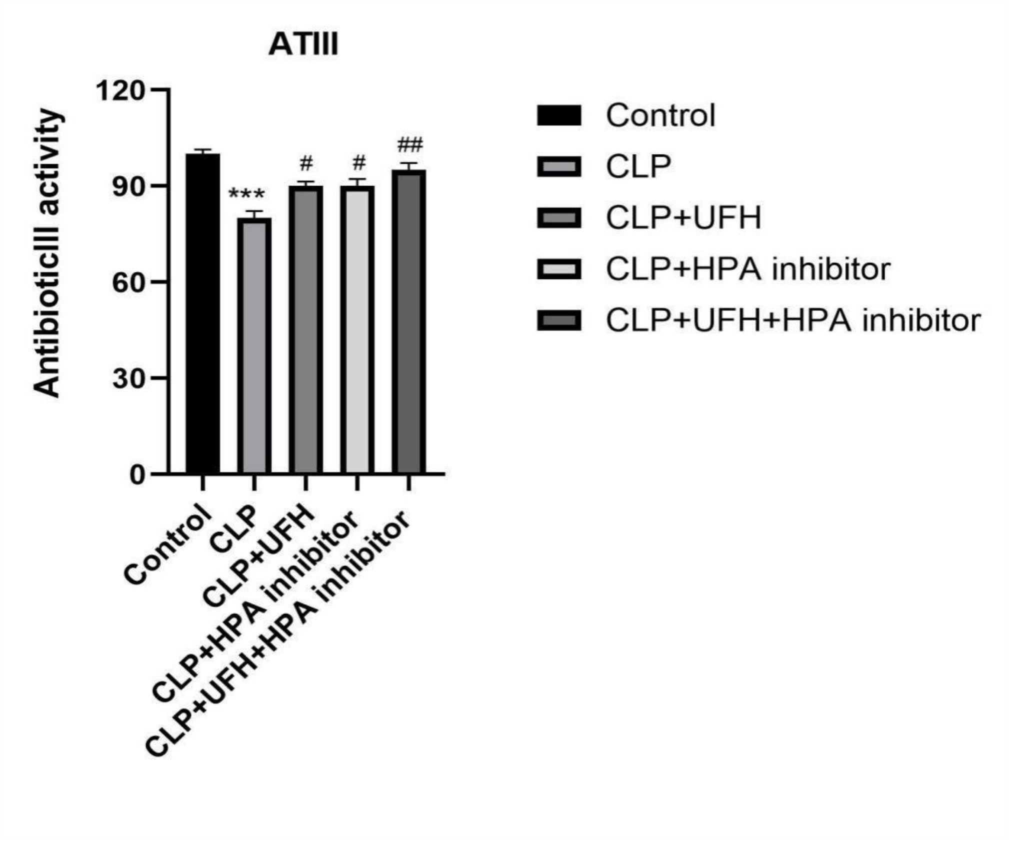

Supplemental Figure 4. Expression of AT-III activity in each group. The AT-III activity in control group is 96-102. The AT-III activity in CLP and CLP+UFH group are 79-87 and 86-98 respectively. The AT-III activity in CLP+HPA inhibitor and CLP+UFH +HPA inhibitor group is 86-95 and 92-103 respectively. UFH, unfractionated heparin; HPA, heparinase. *N* = 6. $^{***}P < 0.001$ *vs.* control group; $^{\#}P < 0.05$, $^{\#\#}P < 0.01$ *vs.* CLP group.

e

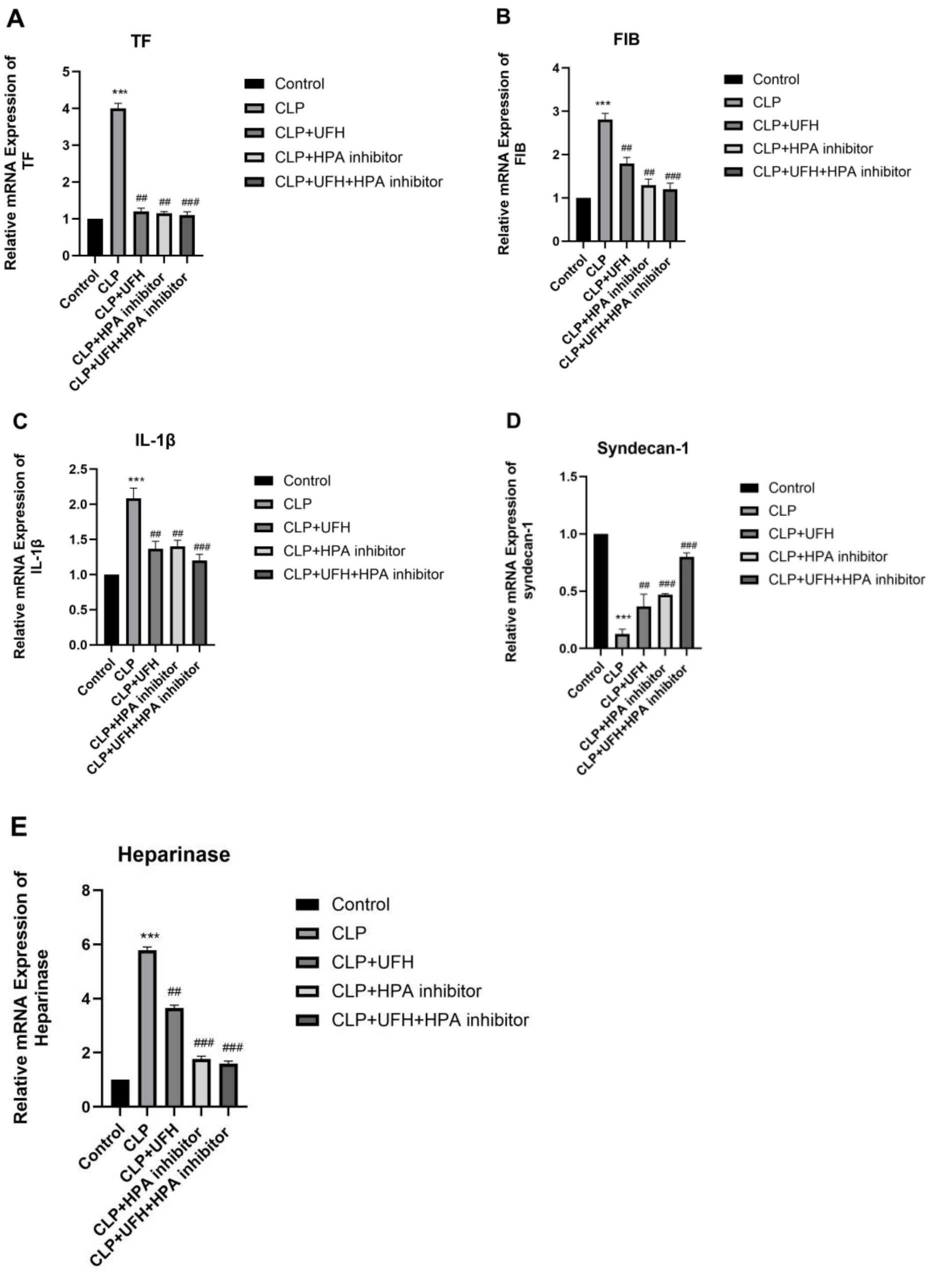

Supplemental Figure 5. The mRNA expression of TF (A), FIB (B), IL-1β (C) Syndecan-1 (D) and HPA (E) in each group. $N = 6$. $^{***}P < 0.001$ *vs.* control group; $^{\#\#}P < 0.01$, $^{\#\#\#}P < 0.001$ *vs.* CLP group. TF, tissue factor; FIB, fbrinogen; IL-1β, interleukin-1β; CLP, cecal ligation and puncture; UFH, unfractionated heparin; HPA, heparinase.

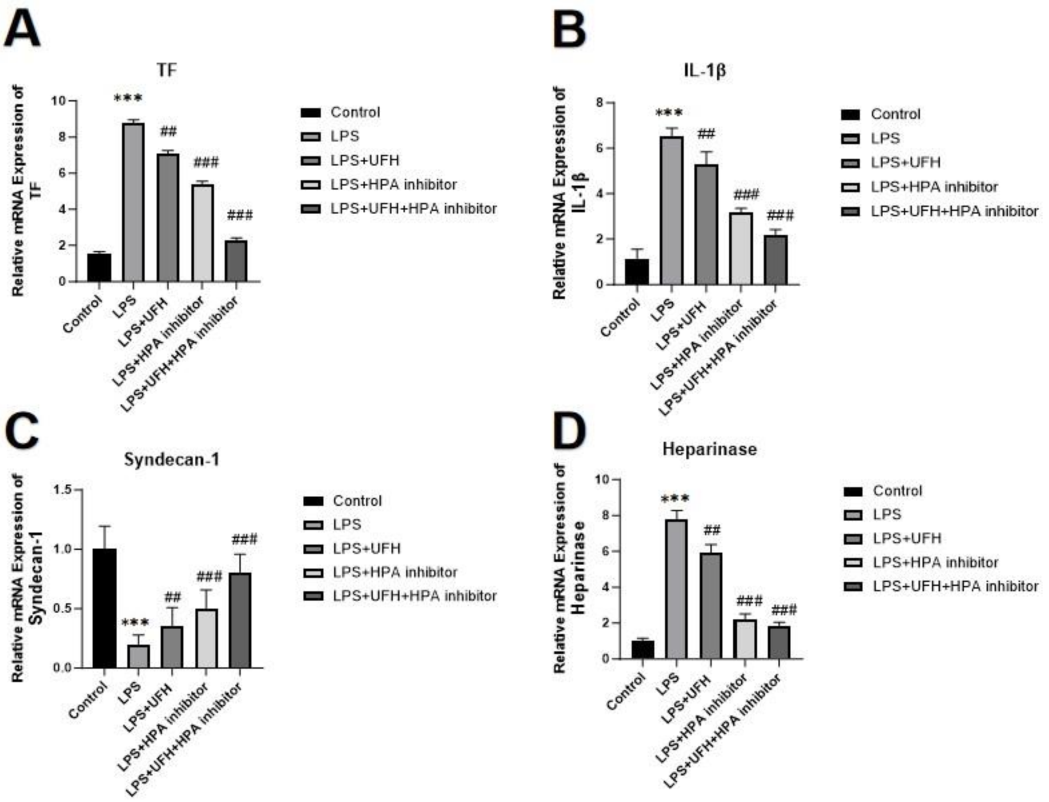

Supplemental Figure 6. The mRNA expression of TF(A), IL-1β (B), Syndecan-1 (C) and HPA (D) in each group. *N* = 6. ***$P < 0.001$ *vs.* control group; ##$P < 0.01$, ###$P < 0.001$ *vs.* LPS group. LPS, Lipopolysaccharide; UFH, unfractionated heparin; HPA, heparinase; TF, tissue factor; IL-1β, interleukin-1β.

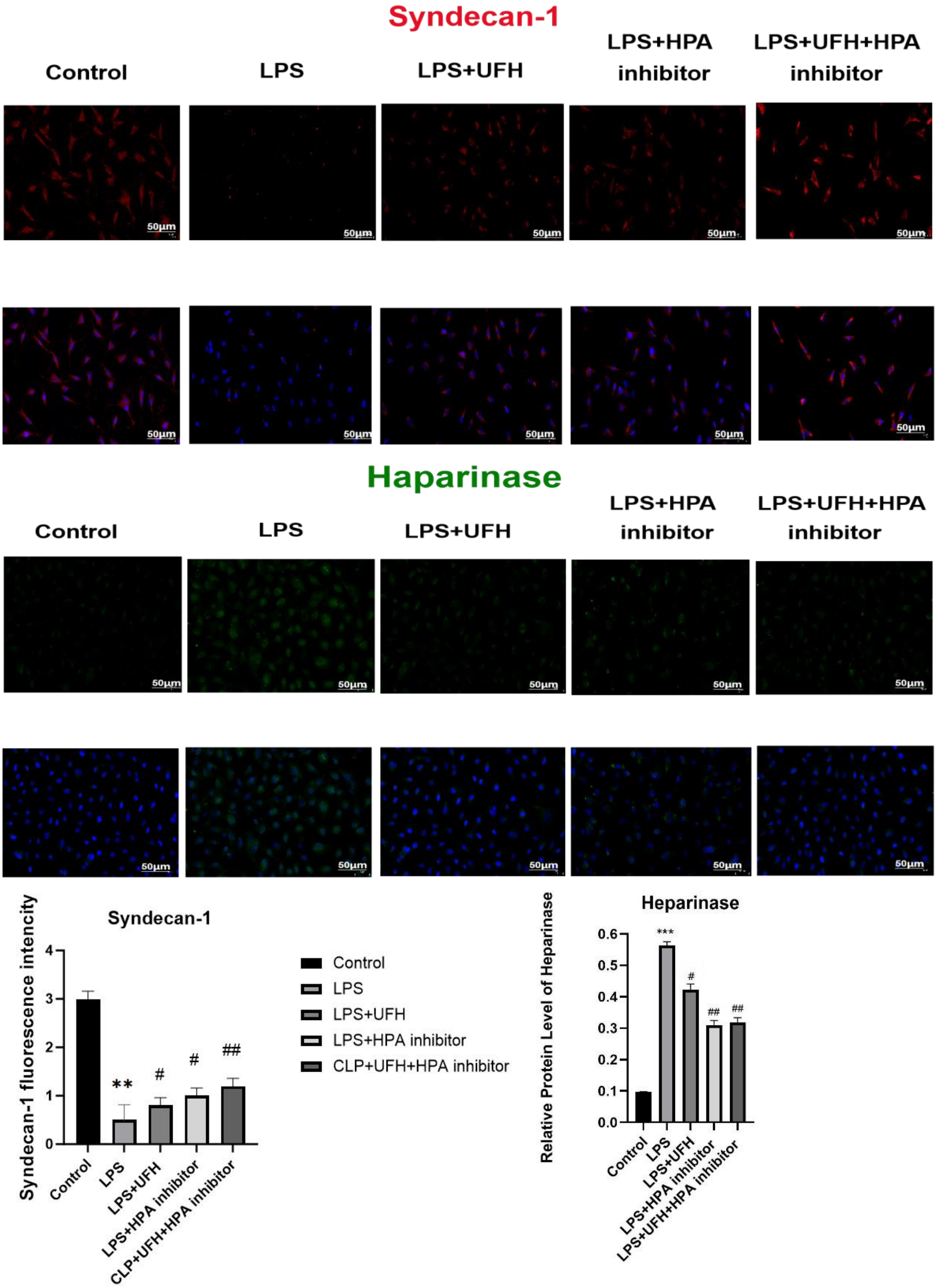

Supplemental Figure 7. The positive expression of HPMECs syndecan-1 and heparinase in each group (200×). Scale bar = 50 μm. $N = 6$. $^{**}P < 0.01$, $^{***}P < 0.001$ *vs.* control group; $^{\#}P < 0.05$, $^{\#\#}P < 0.01$ *vs.* LPS group. LPS, lipopolysaccharide; UFH, unfractionated heparin; HPA, heparinase.

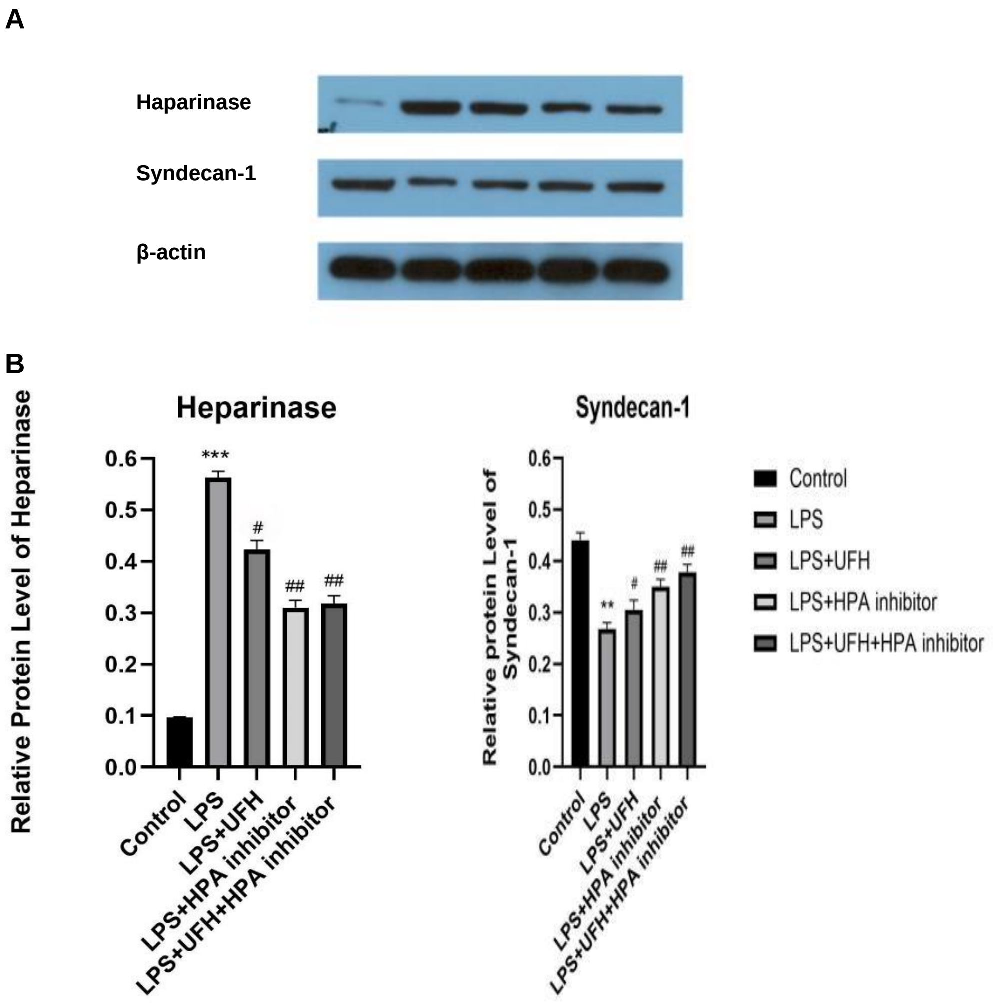

Supplemental Figure 8. The relative expression of syndecan-1 and HPA in each group. $N = 6$. $^{**}P < 0.01$, $^{***}P < 0.001$ *vs.* control group; $^{\#}P < 0.05$, $^{\#\#}P < 0.01$, $^{\#\#\#}P < 0.001$ *vs.* LPS group. LPS, lipopolysaccharide; UFH, unfractionated heparin; HPA, heparinase.
